# Caring for Caregivers of People Living with HIV in the Family: A Response to the HIV Pandemic from Two Urban Slum Communities in Pune, India

**DOI:** 10.1371/journal.pone.0044989

**Published:** 2012-09-13

**Authors:** Rewa Kohli, Vidula Purohit, Latika Karve, Vinod Bhalerao, Shilpa Karvande, Sheela Rangan, Srikanth Reddy, Ramesh Paranjape, Seema Sahay

**Affiliations:** 1 National AIDS Research Institute, Pune, India; 2 Maharashtra Association of Anthropological Sciences, Pune, India; University of Toronto, Canada

## Abstract

**Introduction:**

In low resource settings, the vast majority of ‘Person/people Living with HIV’ (PLHIV/s) and inadequate healthcare delivery systems to meet their treatment and care needs, caregivers play a vital role. Home based caregivers are often unrecognized with limited AIDS policies and programs focusing on them. We explored the perceptions and norms regarding care being provided by family caregivers of PLHIVs in India.

**Methodology:**

A community based qualitative study to understand the issues pertaining to home based care for PLHIV was conducted in urban settings of Pune city, in Maharashtra, India. Eight Focus Group Discussions (FGDs) among men, women and peer educators were carried out. A total of 44 in-depth Interviews (IDIs) with PLHIVs (20) and their caregivers (24), were conducted using separate guides respectively. Data was analyzed thematically.

**Results:**

Home based care was perceived as economically viable option available for PLHIVs. ‘Care’ comprised of emotional, adherence, nursing and financial support to PLHIV. Home based care was preferred over hospital based care as it ensured confidentiality and patient care without hampering routine work at home. Women emerged as more vital primary caregivers compared to men. Home based care for men was almost unconditional while women had no such support. The natal family of women also abandoned. Their marital families seemed to provide support. Caregivers voiced the need for respite care and training.

**Discussion:**

Gender related stigma and discrimination existed irrespective of women being the primary family caregivers. The support from marital families indicates a need to explore care and support issues at natal and marital homes of the women living with HIV respectively. Home based care training and respite care for the caregivers is recommended. Gender sensitive interventions addressing gender inequity and HIV related stigma should be modeled while designing interventions for PLHIVs and their family caregivers.

## Introduction

The annual number of new HIV infections has been steadily declining since the late 1990s and there are fewer AIDS related deaths due to the significant scale up of antiretroviral therapy (ART) over the past few years [1]. Longevity has increased among large number of ‘Person/People Living with HIV’ (PLHIV/s) worldwide [2]. Russel and Schneider opine that this is due to the shift of the silent epidemic of HIV into a visible epidemic of AIDS and hence, ‘care’ and the ‘care agenda’ are getting importance at global level [3]. Both health care professionals and policy makers hence, need to search for accessible health care options that will meet the care related needs of PLHIV and also enhance their quality of life [4].

India has the third largest number of HIV positive individuals [5] and the demand for resources for care is increasing and impacting the health system. Contextual factors such as stigma, discrimination, fear and neglect at the workplace, healthcare settings and in the community and depletion of financial resources have intensified the situation. Care interventions for the HIV epidemic cannot operate in isolation but must be embedded into the spheres of health facility, the community, the workplace environment and also the household. To mitigate the impact of the HIV epidemic there is a need for an integrated and expandable care agenda linked up with the family.

Research has shown that HIV epidemic impinges upon community resources and weighs down caregivers [6]. The review of literature on care provision and HIV by Ogden et al [7] states that family caregivers provide the majority of care to PLHIV. There are many challenges for the ‘family caregivers’ ranging from burnout and financial strain to injury, increased vulnerability to illness and emotional despair [8]–[11]. There may be changes in the family structure and sentiments due to urbanization [12], but the family continues to be a source of strength and support for most people, especially during illness and death [13]. In India, families represent the largest group of caregivers for all chronic illnesses, including HIV [14]–[15]. Kakar [16] reported that the family or the household provides an ideal setting for any intervention because of the existing strong emotional bond that binds members together symbiotically. The caregivers for a PLHIV may belong to a nuclear or an extended family and the family as a whole also takes some responsibility of taking care of PLHIV when he/she is not well and requires care and support.

While assessing any kind of work, economic indicators do not cover the activities of family caregivers, as they do not fall under a monetised economy. This results in disparities between family caregivers and various voluntary groups acting as caregivers. The latter have advantages like recognition, sharing of feelings with other members, getting time-off and having access to support from formal sectors. Moreover, it is a form of duty or job with limited or no emotional attachments. Despite the efforts made globally to improve the care agenda, there are gaps between the formal and informal caregivers that need to be addressed in terms of fulfilling needs of family caregivers. In India, the family members are expected to provide care to any person who is ill in the family and hence, the patient does get care [11]–[13]. However, in the context of care for PLHIV family caregivers are sill not recognised. We conducted a community based study to understand the community’s perceptions and norms regarding the care given by the family members of PLHIV and to examine the challenges that family members and caregivers face on account of home based care.

## Methods

### Ethics Statement

This study was approved by the institutional ethics committees of the National AIDS Research Institute (NARI) and the Maharashtra Association of Anthropological Sciences (MAAS) at Pune respectively. Written informed consent was obtained from the study participants for ‘In-Depth Interviews’ (IDIs) and oral consent from the ‘Focus Group Discussion’ (FGD) participants.

A qualitative study was conducted between 2008 and 2010 in Pune, a high HIV prevalence city of Maharashtra in India. The study participants were recruited from two urban slum communities located in two geographical locations in Pune, with the help of voluntary agencies working with HIV positive individuals. Forty four IDIs were carried out with HIV positive individuals (n = 20) and their caregivers (n = 24) to study individual experiences and perspective of home based care provided by family caregivers. Eight FGDs were conducted with men/women/peer educators from the same communities in order to get the community perspective about care of PLHIV at home. The IDIs and the FGDs were conducted in local ‘Marathi’ language.

### Recruitment

NARI has an established Community Involvement Plan (CIP) for outreach activities in urban Pune through non governmental/community based organizations (NGO/CBO) which are termed as ‘partner NGOs’. Two partner NGOs with acceptance and reach in two urban slum communities were identified for the recruitment of HIV positive individuals for this study. Under CIP, a number of community members have been identified as peers who are trained in human subject research issues [17]. Locally, these peers are under the administrative control of these NGOs and they implement health awareness programs of the NGOs as well as carry out recruitment activities for research being conducted at NARI. These peers were involved for recruitment of study participants for IDIs and FGDs in this study.

### Focus Group Discussions

FGDs focused to understand the concept of care in general and specifically home based care for HIV, were conducted in the community either at a community hall or at the respondents’ residences. A team of four; a facilitator, an observer and two note takers carried out all the FGDs in the local Marathi language. A printed guide and visuals depicting four illness scenarios were used to conduct the FGDs. These scenarios depicted the local socio-cultural context so that the participants could relate with the characters and comprehend the situation shown in these four visuals. The visuals showed different type of illness situations to understand respective concepts of care in the community. The first visual showed a boy with an illness, the second had a bed ridden young man with a fractured leg, the third showed a young man lying on the bed while the fourth one was an illustration of a young widowed woman with a small child on her lap. The facilitator showed each of the visuals to the FGD participants leading to spontaneous discussions pertaining to what care should be given to the person shown in the visual. Then the facilitator also asked open ended questions and used probes to facilitate the discussion on care for PLHIV. Each FGD lasted for 45 to 60 minutes on an average and it was audio taped.

### In Depth Interviews

Purposive sampling was used to cover different categories of heterosexual HIV positive individuals. The PLHIV were selected based on their marital status, age, stages of the HIV disease, belonging to HIV concordant or discordant setting. At the end of the interview, participants were requested to identify their primary caregiver/s with whom IDIs were conducted separately. Separate interview guides were used for PLHIVs and their caregivers respectively. The common questions for both PLHIVs and their caregivers focused on diagnosis of HIV, disclosure, history of the disease and its treatment, behavior of the members of family and social support. Information about the details of illness episodes, the type of care provided by the family, the roles and the needs of the family members as caregivers were collected from the caregivers.

### Data Management and Analysis

Verbatim transcripts of each audio taped FGD and IDI were created and validated by a second round of listening to the tapes. The transcripts were then translated into English. Data for FGDs were coded by one researcher and those of the IDIs by two researchers, ensuring good inter-coder reliability. If there was any lack of clarity or disagreement, it was resolved through discussions to generate new code or add new description to the existing codes. Researchers used Atlas Ti 4.2, the software for qualitative data management to organize and assemble the qualitative data from the FGDs and NuDIST 6.0 for managing the data generated from IDIs. The data from FGDs and IDIs were thematically analyzed [18].

## Results

A total of 8 FGDs were conducted among 88 participants of which 47 were men and 41 were women. Members of youth group, self help group, social workers, peers and housewives participated in these FGDs (Table 1). The age range for men was 20 to 40 years while that for women was 20 to 55 years. Of the 20 PLHIVs participating in the IDIs, 75% were between 25 and 35 years of age, married (75%), with education up to secondary school (75%), had some occupation (65%) and they were staying in nuclear family (85%). All PLHIVs were married among whom one man was separated and four women were widowed. Eight men and three women PLHIVs were on ART (Table 2). Among the 24 caregivers identified by PLHIVs, 18 were women and 6 were men. A total of 9 caregivers were HIV positive and four of them were on ART. Three of the four widowed women identified other relatives as caregivers while one of them did not have any caregiver. One HIV positive man who was separated, reported employer as his caregiver.

**Table 1:** Details of Focus Group Discussion (FGD).

FGD # Group structure	Role in context of community	Gender	Number ofparticipants
**FGD 1** Members of Mitra Mandal(Local youth group)	Providing small financial and social support to PLHIVs	Male	12
**FGD 2** Literate womenwho identified themselvesas housewives	Self Help Group that helped in income generation activitiesand savings among women	Female	11
**FGD 3** Men	Local community members	Male	11
**FGD 4** Social workers	Women working with the families in urban slums and in ruralareas for maternal and child health	Female	11
**FGD 5** Beneficiaries of local NGO	Local community members who had utilized services of NGO	Female	8
**FGD 6** Peer educators	Trained volunteers from the local community who workedin the local community on HIV/AIDS prevention, treatment,research program	Female	11
**FGD 7** Men	Self Help Group that helped in income generation activitiesand savings among men in the community	Male	7
**FGD 8** Unskilled workersemployed in health set up	Local community members with some knowledge of healthcare delivery	Male	17
**Total FGDs** = 08		Males = 47Females = 41	88

**Table 2:** Description of respondents for in depth interview of PLHIVs and their caregivers.

Characteristics of the respondents	PLHIV [N = 20]		Caregivers [N = 24]	
	Men [n = 10]	Women [n = 10]	Men [n = 06]	Women [n = 18]
**Marital status**				
Married	9	6	6	13
Unmarried	0	0	0	2
Widow	0	4	0	3
Separated	1	0	0	0
**Occupation**				
Housewife	0	5	0	7
Unskilled worker	9	5	0	8
Peer	0	0	0	1
Skilled worker	0	0	6	1
Student	0	0	0	1
Unemployed	1	0	0	0
**HIV status**				
HIV positive	10	10	4	5
HIV negative	0	0	2	13
**ART status**			n = 04	n = 05
On ART	8	3	3	1
ART naive	2	7	1	4

Nearly half of the HIV positive men disclosing high risk behavior attributed it to the nature of their job, sufficient money to splurge on eating, drinking at bars, visiting sex workers etc. It was revealed that they were ignorant about the use of condoms. Some of them denied having high risk behavior. ‘Blood’ was stated as another cause by some of the participants; in some cases women tried to give it as alternate explanation for the HIV infection of their spouses.

An HIV positive man (CS04), whose caregiver was his spouse, said, *“There are many things… once or twice I met with accidents [/had bled and believed it as probable reason for acquiring HIV/] and once or twice I had gone ‘there’ [/brothel/] with my friend”*. Similarly a married HIV negative woman caregiver (CS05) said: *“My ‘mister’ told me that he had gone out to one of the ‘ladies’ [/female sex worker/]. He had relations there. There he had a wound and bleeding”*.

Two women stated that their spouse may have become positive through injection; one mentioned injury to his hand in an accident to be the cause and another one said she did not know the cause. Four women could explicitly state that they were HIV positive due to the high risk behavior of their spouses but they learnt this only after the death of their spouses. The route of HIV infection among men, however, did not affect the care provided by HIV positive or negative women in their families. Majority of the PLHIVs (65%) and their caregivers (63%) had mentioned economic difficulties.

All PLHIVs reported deterioration of their health after acquiring HIV infection, with episodes of intermittent illnesses. Responding to the four visuals in the FGDs, most of the FGD participants felt that the family members should take care of their family member in case of any illness whether it is *“a minor illness like cough, cold or fever or a severe illness like TB, HIV or paralysis”.* The caregivers seemed to have taken the responsibility for many aspects of care. But they said that there was no formal or informal support for the family caregivers. The data was further analyzed to understand issues pertaining to the concept of care being given by the family caregivers of PLHIVs. The emerging themes from the study were: 1. Home based care 2. What comprises home based care? 3. Gender in family care 4. Needs and preferences of the family caregiver.

### Home Based Care

Half of the PLHIVs (10/20) and their caregivers (13/24) preferred home based care owing to affordability, free of stigma and convenience. In the focus group discussions, people opined that a lot of energy is spent on taking care of the patient at home while hospital based care was costlier. The commonly stated expenses were transportation, associated expenses for food for caregiver and/or for the patient and hospital charges. In addition, caregivers faced a lot of physical hardship as there was no place for them to sit or relax in the hospital. A woman caregiver (CS06-CG01) explained, *“Expenses will always be there, additionally I have to wake up early to prepare food, to take tiffin (packed lunch) to the hospital, serve him food and stay in the hospital. There will be traveling expenses also”.* Women from the community (FGD 002) also felt that cost should be considered and unless the condition of the patient requires hospitalization, he/she should not be hospitalized. *“Money is not enough [/low economic status/] to manage everything. Even if we have to help her, we do not have the treatment facility [/hospital/]) where such a patient [/patient from low socio-economic strata/] can be admitted forever [/for long periods/]. We can instead, treat the patient at home by giving him/her adequate care and support. This way we can reduce their burden also and help them”.*

One of the underlying reasons for avoiding hospital also seemed to be discriminatory experiences by the hospital staff. Cost also escalated due to some of the practices in the hospital as shared by a 32 year old married ART naïve HIV positive woman (CS-18), *“When his kidney stone operation was done in = name of hospital = , then there they [/doctors/] did the test [/HIV test/]. Then they asked to pay more bills”.*

*Then I asked, “What’s that for? This much you had demanded [/earlier/], and then how come you are asking for more”?*

*They [/Hospital staff/] said, “We have to throw all the stuff [/instruments/], it happens because of this [/HIV/]”.*

Some of the caregivers reinforced that there is no fear of stigmatization and disclosure in case of PLHIV receiving home based care. Fear of stigma surfaced, as some of the women caregivers visualized neighbors coming to know about the HIV: *“When even the topic [/of HIV/] is raised, their [/neighbors’/] point of view is different and … suddenly you can see the hatred in their mind. Then the thought comes to our minds that if they [/(neighbors/] come to know of our illness they will behave in the same way with us… like this [/fear shown by action/]”.*

Since the majority of the family caregivers were women, they also preferred caring at home as they were able to manage income generation and household responsibilities efficiently with assistance from neighbors. Assistance from children was also reported to be useful in case of emergency. Besides, the patients preferred to remain in their family environment where friends and entertainment facilities were readily available. Home based care was discussed by both communities and PLHIVs as having the advantages of extra care and personal attention for meals/medicines which is not possible in a hospital setup.

Women living with HIV, on the other hand, seemed to prefer ‘hospital based care’ for themselves owing to satisfactory services that they would receive at hospital as compared to home. An HIV positive widow (CS13) said, *“In the hospital everything is on time, timely breakfast at 10 means 10, that never happens at home. Lunch at 12 O’ clock… At 4 pm tea-biscuits that is the difference between home and hospital.”*

### What Comprises Home Based Care?

The family caregivers give emotional support to PLHIVs and also provide relaxation. At home, care of the patient included taking care of basic needs, maintaining optimal level of hygiene becomes possible for them. The facilities of medication, meals, advice to take rest, facilitating periodic blood tests and giving medicines on time are ensured conveniently. Women from the community (FGD002) threw light on the process of family care giving*: “Give meals and medication on time. Discourage the patient from going out/moving in hot weather”.*

Most of the family caregivers were constantly alert about adherence and compliance to the treatment. An HIV positive ART naïve woman caregiver (CS12-CG01) described care inclusive of adherence as follows: *“Care means giving medicines on time, telling him to go to the hospital, giving food on time because he needs to take medicine on time. I always enquire whether medicine has been taken or not. He takes his meals to work so I also telephone him to check if he has taken the medicine on time”.*

All the caregivers reported having received counseling for special nutritional needs of the HIV positive individuals, like giving leafy vegetables, pulses and fruits. Adhering to this advice in order to maintain the general health of PLHIV and meeting special dietary needs during illness episodes resulted in most of the money being spent on food items leaving little or no money for other expenses. Community members, based on the experience of the cases they had witnessed, felt that since caring for an HIV positive individual is a tedious and a difficult job; caregivers would feel depressed with no hopes about the future and would be tired of taking care of PLHIV and living in fear of getting infected. This was borne out by one of the respondents, where a woman caregiver had left her home as she had become tired of taking care of her ailing HIV positive spouse.

The caregivers cared about the quality of life of PLHIVs, sometimes at the cost of their own emotional health. Mother of an HIV positive man (CS02-CG02) said, *“I do not cry in front of my son. I cry when nobody is there in the house”.* Some women caregivers did not talk or question the HIV positive person about HIV at all. Caregivers made efforts not to add any mental or emotional stress to the positive individuals. They asked them to take rest, watch television etc. to divert their minds away from their illness. Spouses avoided picking up any argument with their HIV positive male partners. They never enquired about the source of infection from their partners and remained silent even if they were aware of their infidelity.

If the family caregiver was HIV positive, he/she would also be motivated to protect other members of the family. They took precautions to prevent HIV transmission through injuries, cuts or wounds. It was observed that their family members had misconceptions. They often asked the positive person to keep children away from them; some positive women were also prevented from undertaking jobs which involved cutting and chopping in the kitchen. In the FGDs, the understanding about HIV transmission was evident among a certain set of people in the community such as peers and social workers residing in the same location. For example, peers (FGD 006) summarized their experiences as follows: *“We know [/have knowledge/]. We don’t have fear of HIV. We will not get [/HIV/] by touching him; that’s why we help. When we go in field area, if such person is there, we sit beside him, eat in the same plate”.* But the practices of an ART naïve widow (CS13) were as follows: *“Now if I ask her [/daughter/] for a glass of water, then I don’t let her drink from the same glass. I don’t let her touch it at all. The water that I’ve drunk, I do not allow anybody to have it. Now I do not share the food from my plate with anybody else at home”.*

Practice to prevent sexual transmission of a male PLHIV (CS05) was shared, *“I use condom. After using ‘Nirodh’ [/Brand of condoms distributed by government of India free of cost/] if there is a ‘wish’ [/for sexual intercourse/]’, then I have no problem, I use, not one but two condoms …She is ‘negative’ till now [/He believes that his spouse has not acquired HIV up till now because he has used double condoms/].”*

As compared to women, HIV positive men provided limited support to their HIV positive or negative spouses. For example, an HIV positive woman said that her positive husband stayed at home only when he was unwell but not when she was unwell and needed help at home.

### Gender in Family Care

Women emerged as the primary caregivers and this was also borne out by the data from the in-depth interviews; 75% of the caregivers were women (18/24). Community expectations for care also reflected that women, especially spouse, were the primary caregivers for PLHIVs.

FGD 002: *“His wife will take care… Yes, the wife will have to take care. She will only take care”*.

Men emphasized that HIV infection is transmitted through four modes only; hence, woman should take care of HIV positive persons without any problem. Male family members such as the father or the brother provided financial support and accompanied the person to the clinic, but did not share the household chores. Close relatives like parents, wife’s parents, siblings, etc. were expected to pay visit to the patient.

Women caregivers in the family shared that they had to do multitasking and finish all the activities within a time frame and also to attend the patient’s needs. Men (FGD 005) also confirmed that women caregivers did multitasking, *“Along with that person [/patient/] she has to take care of family members also. More stress will be on her. To look after that person by finishing work of house, care of taking him to the hospital, hygiene, these entire things she has to do”.* Other women if present in the family would help the caregiver by sharing some household activities like fetching water and washing utensils. A female social worker in a focus group said, *“If there was a young daughter then she would cook, she would clean the utensils otherwise cooking for family members is must, in spite of the illness [/of the woman/]”*.

Spouses of HIV positive men expressed their inability to support themselves for outdoor work. Most of the women in this study were housewives (12/28) while others were doing odd jobs such as housemaid, rag picking, pottery or tailoring etc. One of the caregivers was a peer educator while another woman caregiver was a student. An HIV negative spouse (CG08) of HIV positive man said, *“Now [/you see/] a woman can’t do anything in front of a man [/can’t take any decision without a man/], cannot go anywhere for work… we [/I/] don’t know anything first of all; he brings the entire ration etc. I don’t know about outdoor work also, how to bring vegetables. I don’t know anything. He goes to bring the children from school in the morning, and then he brings vegetables. And I don’t know at all about outside work”.* The women in the community (FGD 002) opined that it is the woman’s responsibility to take care of her spouse and keep him alive. Women provided unconditional support to their HIV positive spouses even if it meant having sexual relationship. An HIV negative woman (CG05) in HIV discordant setting shared, *“Support? I never say anything about his illness. We have no disputes because of his illness. I never tell him to do this or don’t do this. That you have this illness! We have no disputes, no fights over the physical [/sexual/] relationship”*.

Care for men living with HIV was perceived by women as vital because they needed to gain health and start earning again. Hence other family members were also never averse to provide them care and support even when the resources were limited. On the other hand, in case of women living with HIV, family support was very limited. A 29 year old HIV positive woman (CS02-CG01) who was also the caregiver of her HIV positive spouse told about her situation at home, *“I am not staying with them [/family/], since last six months, because now their harassment is beyond my tolerance. That is why I thought that it is better to live separately. For the last 6 months, I have been living separately in the attic of our house. I was not well for two days. I would take bath but could not wash my clothes. For two days, my clothes were just lying there. On the third day, I came down, somehow managed to wash those clothes and dried those…. Others [/family members/]….they are good with him [/HIV positive spouse/]. They never stopped him from moving anywhere in the house and interacting with anyone.*

Another 32 year old HIV positive woman (CS18) in a similar HIV concordant setting shared about the treatment meted out to her by the family.

*She [/family member/] said, “We will get ‘this’ [/HIV/] because of you. You sit outside [/you menstruate/]. My son doesn’t have anything like it but you menstruate so we can get ‘it’ [/HIV/] from you”.*

Women were the primary caregivers in the family. These women reported seeking health care at formal setting. An HIV positive ART naïve woman caregiver (CS06-CG01) shared, *“Then if I have my needs, responsibilities, have to fulfill myself only. If I fall sick or if something happens, have to go to hospital and take injection myself only. I don’t tell anybody. I don’t tell anybody that I am sick”.* Women living with HIV preferred to go to the hospital to seek health care and they were quick to seek help as an HIV positive married woman (CS17) advised, *“We should take care that if we ever get hurt, or have fever, or chills then immediately go to the hospital and take medicines or pills. Don’t let it progress”.*

Isolation, need for care and affection were observed among women living with HIV because their natal family abandoned them. Only few women living with HIV (2/10) said that their natal family supported them. A 28 year old HIV positive widow (CS01) narrated her situation after her husband passed away, *“I got married. After 1½–2 years, he came to know that such and such disease [/HIV infection/] he has. At that time my father had taken me [/to my parents’ house/] for not getting HIV infection to me but I came to know that I also had this disease at medical [/hospital/]. Then he [/my father/] dropped me back [/to my husband’s house/]. Mother and father kept me at some distance but mother in law and father in law did not do this. She [/mother/] asked me to come at my home [/natal home/]? Come to live? Never! (Cried)… Nobody used to call for program [/natal family function/]”.* Another 39 year old HIV positive ART naïve widow (CS13) shared the similar story of rejection by the natal family as follows. *“Because they came to know about [my] disease [HIV infection], so even they [/parents/] rejected me, my brother & brother’s wife all of them”.*

Many women living with HIV (6/10) reported receiving support from women in their marital family. A 65 year old widow caregiver (CS01-CG01) of a young HIV positive widow explained, *“Taking care is must. Now we are only there for each other. I am really concerned about what will be her future after my death. I look after her and she takes my care. She looks after the house. She got “this” [/HIV infection/] because of my son, so I need to look after her”. (She cried).* Community (FGD 004) also observed that the ‘widowed women living with HIV’ received support from women in their marital homes: *“Her mother in law…. She had lost her adult son. And the daughter in law … she lost her life partner. They don’t have anybody’s support. They are helpless. Both of them are giving support to each other. The daughter in law has her son’s responsibility and she also has the illness”.*

### Needs and Preferences of Family Care Givers

FGD (006) participants stated *“Everybody is living in the same socio-economic situation.”* And this was an indication of the monetary needs of the families because HIV is associated with morbidities hence work absenteeism and loss of work. Getting job or going back to work especially to take care of children was the expressed need of both PLHIVs and their caregivers. An HIV positive ART naïve caregiver spouse (CS11-CG01) clarified her need, *“I am not talking about the support from other person…. I mean to say that if someone gives me support for work… gives me some work; then keeping my children in the hostel looking after them… I will feel very satisfied”.* Men in the community (FGD 003) also felt financial support should be given to families of PLHIVs. They suggested, *“If the financial condition of the family is very poor then some institutions or organizations supporting such cause can help. May be the elected member in the community will be able to help”.* Participants in almost all FGDs were vocal about the help required.

FGD 004: *“In these days of price hike getting food for us is also very difficult…How long people can help? Not like that. If any organization or even the government gives the wheat at low rate…”*

Discussion with community members revealed that there were many voluntary agencies providing diverse services to HIV positive individuals in the locations where the study was conducted. These services included awareness generation and provision of support. They imparted basic information on HIV and AIDS, its treatment and also highlighted the significance of adherence and home based care. Some agencies were providing services like giving medicines, nutritional, educational and financial support as shared by an.

HIV positive woman (CS13) beneficiary, *“They = name of an NGO = also give tablets [/medicines/]. They used to give me ration, first to me, and then for my daughters also. Every Monday they used to give me small amounts… Jowar (a millet) 1kg, wheat 1kg.*

*“Children’s expenses… we get it from… this NGO. It is from them … we get the children’s school expenses for all the three children”* [As shared by an HIV positive man (CS15)].

Nearly half of PLHIVs had utilized the services of these NGOs. Few of the affected and HIV positive women had also begun working as peers with some of these organizations.

The need for training in home based care was also expressed. A 27 year old married HIV negative caregiver woman (CS05-CG01) said, *“Somebody should give me information. Now I am taking his care according to the information I have. If somebody tells me something more or if I get some information then I immediately try it out the next day. If somebody teaches me a new food recipe and tells me that it gives plenty of vitamins, then I immediately make the new dish and give him to eat”*.

Another respondent wanted training in home based nursing care. Respondents suggested that this training could be in the form of a lecture or through audio-visual media as was previously done by some NGOs.

## Discussion

The primary goal of the study was to elicit explanations of care of PLHIVs in family settings as perceived by the community, PLHIVs and their caregivers in India. Most of the caregivers were women, mostly spouse and common scenario was that in the families with PLHIVs, spouses were also often HIV positive. The findings in this study reflect the issues of care being provided by family caregivers especially women with its associated burden, stigma and gender inequity in receipt of care and care provision. The family emerges as the major caregiver institution for PLHIVs in India as observed in other developing nations [19]–[21]. Women emerged as the primary family caregivers as has been reported in several studies from Uganda and South Africa including India [7], [11], [22]–[24]. The hierarchy of care follows a typical gender pattern in the family with the spouse of HIV positive man expected to be the foremost family caregiver irrespective of her own HIV status and related needs. This is followed by other women in the household i.e. sister, sister in law and mother in law respectively. Women living with HIV seemed to receive some support from marital families as compared to their natal families who invariably abandoned HIV positive women which needs to be explored in India. One of the limitations of this study is that it only includes PLHIVs who had heterosexual orientation and thus findings cannot be generalized to all types of PLHIVs.

Both PLHIVs and their caregivers preferred home based care in this study. In other countries like Uganda, family care giving was the most common form of care for PLHIVs which was often due to access and costs related issues pertaining to clinic based care [25]. However, preference for home based care observed in our study was because it was convenient personalized family care for PLHIV and it was easy to maintain confidentiality within the family set up. The associated cost saving on commuting and food that one buys if a patient is hospitalized, facilitating work from home etc. altogether made home based care also economically more viable option for the families. Need for employment and financial support emerged among the caregivers and women preferred home based care to fulfill this need. It enabled them to manage income generation from home without hampering their routine household activities.

A critical appraisal of the emerging themes in context of stigma and positioning of gender might help in understanding the issues of home based care of PLHIVs in India. Our study shows that communities are still struggling with stigma although many trained group of community members lend voice to the fact that HIV related stigma has reduced. However, the gap exists. The emerging preference for home based care was because of the fear of disclosure in the community in case of hospitalization of PLHIVs. Apparently, when the community talked about care and support, it seemed as though HIV related stigma did not exist, yet PLHIVs, especially women and families of PLHIVs were concerned about stigma which often led to isolation of PLHIVs and their families. In India where cultural system places greater emphasis on collectivism, HIV/AIDS might still be perceived as bringing shame on the family and community [19], [26]. Typically, the women caregivers put forward alternate explanations for the HIV infection among their male relatives, ignoring infidelity as reported in other studies where women kept silent to avoid stigma [27]. Additionally not asking questions about cause of infection and total dependence on spouse were some of the typical expressions of internalized oppression among women of ‘being weak/unworthy/no right to be angry’ [28]–[29].

Overall, the situation of women living with HIV in this study reflects existing gender dynamics in the country. We can use oppression framework proposed by Amaro and Raj [28] to understand conditions and behaviors of women living with HIV both as patients as well as the family caregivers in India. The framework of oppression comprises of ‘silencing, violence and fear of violence and internalized oppression’. Owing to the fact that generally a woman gets HIV infection through her marriage in India [30], an HIV positive woman would carry the dual burden of being ill herself and being a caregiver of the spouse who would invariably be in the advanced stages of illness, resulting in ‘silencing’ and ‘internalized oppression’ components as evident in our study. The culturally acceptable norm of a woman being the caregiver irrespective of her own health, is the precise evidence of ‘culture’ and ‘knowledge’ being defined in terms of men’s experience in the society [31]. Woman being a silent caregiver stresses that power in the context of oppression includes prescription of acceptable behavior that is defined by the oppressor [31]–[32]. The women caregivers in this study were the positive stereotypes of women; helpful, gentle, kind and understanding [33] but it is also a burden to carry the stereotype life long. Women caregivers have already reported multiple problems of psychological, social and economic significance in providing care to PLHIV in their families [27]. If women as a group reflect and analyze their own stereotype critically, it might not be acceptable to them.

The hyper-vigilance observed among HIV positive women caregivers emphasize their need to remain healthy essentially to discharge multiple responsibilities towards household. Stigma and discrimination of women living with HIV have been previously reported [14]. In our study too, the discrimination that women living with HIV faced in their families left them disadvantaged which furthered their vulnerability to stigma and discrimination by the family. In turn, it disabled them from challenging their own situation and they did not expect to receive home based care despite being the primary family caregiver for the male PLHIV in the same family. As a result, their own HIV status led to discrimination from their family reinforcing the result of interaction between diverse pre-existing sources of stigma and discrimination such as gender, fear of contagion and disease [34]. Thus, we observe that women living with HIV preferred formal care for themselves instead of home based care. Stand alone empowerment programs for women may not bring any social change. Women can be brought together as a group and interact to develop community based initiatives and projects to bring social change aiming at gender equity in HIV care in families. Women empowerment programs involving HIV concordant and discordant couples focusing on home based care should be developed. These programs should also focus on the specific needs of widowed women living with HIV.

Although an important group, family caregivers are not recognized by the formal sector, which leads to their unaddressed needs of training in nursing care, dietary requirements of PLHIVs and their own psychological needs, physical support and need of a reliever. The operational guidelines for care in community centre under the national program includes training in home based care [35]. The WHO care continuum addresses these needs and suggests better linkages between family caregivers and other care groups to empower them to be caregivers [36]. Developing home based care models of supportive environments, promoting mental health and nutrition, respite care, socio-economic support and integrating community based care into the existing continuum of care might be useful [37]. Scaling up of the home based care program in India viz. models based on local health care providers [38], faith based organizations [39] and community health workers [40] is recommended. These existing models have, however, not focused on the needs of family caregivers. The respondents were beneficiaries of several local voluntary organizations. The voluntary organizations can be scaled up to address the capacity building and respite care needs of family caregivers. The PLHIVs in this study demonstrated motivation to prevent secondary transmission but there was knowledge gap which needs to be addressed by the program. Program for HIV prevention should be expanded to include PLHIVs.

With the advent of free roll out of ART, the life of PLHIV has prolonged [41], acknowledging this sector of caregivers now becomes an important imperative. We suggest gender empowerment interventions for family caregivers which should be able to address gender equity and HIV related stigma.

### Conclusions

Home based care was found to be an accessible and affordable option available to PLHIVs in India. Family caregivers also need to be supported both from the government and voluntary agencies in building their capacities for home based income generation with occasional assistance in care provision. This study acknowledges gender differences among family caregivers of PLHIVs bringing forth the need for interventions to include both men and women as equal partners in care giving. Avenues of care for women living with HIV should be explored further. Comparative studies to understand care and support from their natal and marital family should be planned. No formal agency would be able to take up the cause of life long care except family caregivers and our study brings out the gaps that can be plugged to make family care institution an important pillar for HIV care and treatment.
